# Влияние диеты с повышенным содержанием жира на липидный профиль ооцитов мышей

**DOI:** 10.18699/VJ20.645

**Published:** 2020-08

**Authors:** E.Yu. Brusentsev, E.A. Chuyko, K.A. Okotrub, T.N. Igonina, I.N. Rozhkova, D.S. Ragaeva, S.V. Ranneva, V.A. Naprimerov, S.Ya. Amstislavsky

**Affiliations:** Institute of Cytology and Genetics of Siberian Branch of the Russian Academy of Sciences, Novosibirsk, Russia,; Institute of Cytology and Genetics of Siberian Branch of the Russian Academy of Sciences, Novosibirsk, Russia, Novosibirsk State University, Novosibirsk, Russia; Institute of Automation and Electrometry of Siberian Branch of the Russian Academy of Sciences, Novosibirsk, Russia; Institute of Cytology and Genetics of Siberian Branch of the Russian Academy of Sciences, Novosibirsk, Russia,; Institute of Cytology and Genetics of Siberian Branch of the Russian Academy of Sciences, Novosibirsk, Russia,; Institute of Cytology and Genetics of Siberian Branch of the Russian Academy of Sciences, Novosibirsk, Russia,; Institute of Cytology and Genetics of Siberian Branch of the Russian Academy of Sciences, Novosibirsk, Russia, Novosibirsk State University, Novosibirsk, Russia; Institute of Cytology and Genetics of Siberian Branch of the Russian Academy of Sciences, Novosibirsk, Russia, Novosibirsk State Agrarian University, Novosibirsk, Russia; Institute of Cytology and Genetics of Siberian Branch of the Russian Academy of Sciences, Novosibirsk, Russia, Novosibirsk State University, Novosibirsk, Russia

**Keywords:** mice, diet, oocytes, intracellular lipids, Nile Red, confocal laser scanning microscopy, Raman spectroscopy, мыши, диета, ооциты, внутриклеточные липиды, нильский красный, конфокальная лазерная сканирующая микроскопия, спектроскопия комбинационного рассеяния света

## Abstract

Существуют предпосылки того, что у женщин с ожирением возможно снижение качества ооцитов. При этом остается неясным, как связано это изменение с ожирением: опосредованно или напрямую, че- рез изменение содержания и/или состава липидов в ооцитах. Целью настоящей работы было изучение на мышах влияния богатой жирами диеты, применяемой к самкам-донорам, на качественный состав и общее количество липидов в незрелых и созревших in vivo ооцитах. Установлено, что диета, богатая липидами, при- водит к увеличению массы тела самок мышей по сравнению с контролем ( p < 0.001; 44.77 ± 1.46 и 35.22 ± 1.57 соответственно), а также уровня холестерина ( p < 0.05; 2.06 ± 0.10 и 1.78 ± 0.10 соответственно) и триглицери- дов ( p < 0.05; 2.13 ± 0.23 и 1.49 ± 0.21 соответственно) в крови этих животных. Эта диета не повлияла на степень ненасыщенности внутриклеточных липидов незрелых (0.207 ± 0.004 в эксперименте и 0.206 ± 0.002 в контроле) и зрелых ооцитов (0.212 ± 0.005 в эксперименте и 0.211 ± 0.003 в контроле). При созревании ооцитов in vivo на- блюдалось возрастание содержания внутриклеточных липидов. В зрелых ооцитах количество липидов было больше в экспериментальной группе по сравнению с контролем ( p < 0.01; 8.15 ± 0.37 и 5.83 ± 0.14 соответствен- но). Выявлено увеличение количества внутриклеточных липидов при созревании ооцитов как после стан- дартной диеты ( p < 0.05; 4.72 ± 0.48 и 5.83 ± 0.14 соответственно), так и после диеты, богатой жирами ( p < 0.001; 3.45 ± 0.62 и 8.15 ± 0.37 соответственно). Таким образом, при созревании ооцитов мышей in vivo возрастает со- держание внутриклеточных липидов, богатая жирами диета приводит к повышенному содержанию липидов в зрелых ооцитах.

## Abstract (en)

There are evidences that obese women exhibit a detrimental oocyte quality. However, it remains unclear how this change is associated with obesity, indirectly – or directly through a change in the content and/or composition of lipids in oocytes. The aim of this work was to study effects of a high-fat diet applied to female donor mice on the amount and qualitative composition of lipids of immature and in vivo matured oocytes. A high-fat diet caused larger body weight in female mice compared with the control ( p < 0.001; 44.77 ± 1.46 and 35.22 ± 1.57, respectively), and increased the blood levels of cholesterol ( p < 0.05; 2.06 ± 0.10 and 1.78 ± 0.10, respectively) and triglycerides ( p < 0.05; 2.13 ± 0.23 and 1.49 ± 0.21, respectively). At the same time, this diet does not affect the level of unsaturation of lipids in immature (0.207 ± 0.004 in the experiment and 0.206 ± 0.002 in the control) and matured oocytes (0.212 ± 0.005 in the experiment and 0.211 ± 0.003 in the control). Total lipid content increased during in vivo maturation of mouse oocytes. The amount of lipids was greater in mature oocytes in the experimental group compared to the control ( p < 0.01; 8.15 ± 0.37 and 5.83 ± 0.14, respectively). An increase in intracellular lipid amount during oocyte maturation was revealed both after a standard diet ( p < 0.05; 4.72 ± 0.48 and 5.83 ± 0.14, respectively) and after a fat-rich diet ( p < 0.001; 3.45 ± 0.62 and 8.15 ± 0.37, respectively). Thus, during in vivo oocyte maturation in mice the content of intracellular lipids enhanced, the high-fat diet aggravated this dynamics of lipid increase during in vivo maturation of oocytes.

## Введение

Ооцит и клетки кумулюса, составляющие кумулюс-ооцит- ный комплекс (КОК), содержат липидные гранулы (ЛГ), которые связаны с другими органеллами, участвующими в клеточном метаболизме (Kruip et al., 1983; Dunning et al., 2014; Ellenrieder et al., 2016). Предыдущие исследова- ния, проведенные при помощи световой и электронной микроскопии, выявили связь между эндоплазматическим ретикулумом, митохондриями и ЛГ в ооцитах крупного рогатого скота; эти кластеры назвали метаболическими единицами (Kruip et al., 1983). Тесная связь между этими органеллами способствует клеточному метаболизму, в частности β-окислению липидов (Ellenrieder et al., 2016). Цитоплазматические ЛГ являются хранилищами жиров, которые могут быть использованы в качестве энергетического субстрата (Thiam et al., 2013). Гидрофобное содержимое этих гранул, состоящее в основном из триацил-глицеридов и сложных эфиров стеролов, таких как хо- лестерин, окружено монослоем фосфолипидов (Walther, Farese, 2009). В настоящее время ЛГ считают активными внутриклеточными структурами, играющими важную роль в клеточном гомеостазе (Walther, Farese, 2009; Welte, Gould, 2017). Кроме того, недавние исследования показа- ли их протекторную, а также регуляторную функцию, в частности их участие в белковом метаболизме и работе ядра (Welte, Gould, 2017).

Влияние ожирения на качество ооцитов – важный ме- дицинский аспект. Избыточная масса тела отрицательно сказывается на репродуктивном здоровье людей, о чем свидетельствуют клинические данные, полученные при применении вспомогательных репродуктивных техно- логий – ВРТ (Robker, 2008; Souter et al., 2011; Dickey et al., 2012). Установлено, что у женщин с ожирением на- блюдается снижение качества ооцитов в циклах ВРТ по сравнению с имевшими нормальный вес тела (Robker, 2008). Между тем не ясно, связано ли это с избыточной массой тела пациенток либо с изменением содержания и состава липидов в ооцитах (Pantasri et al., 2015).

Эффект обогащенной липидами диеты на развитие ооцитов подтверждается и в экспериментах на различных видах животных (Zeron et al., 2002; Minge et al., 2008; Wu et al., 2010; Dunning et al., 2014). Так, в работе на мышах, в которой самок держали на диете с повышенным со- держанием жиров, продемонстрировано, что у особей с вызванным таким питанием ожирением было низкое качество ооцитов, а эмбрионы из них хуже развивались в культуре in vitro (Minge et al., 2008). Однако до сих пор остается неизвестным, что именно вызывает эти измене- ния. Цель нашей работы – изучение на мышах влияния богатой жирами диеты, применяемой к самкам-донорам, на качественный состав и общее количество внутрикле- точных липидов незрелых и зрелых ооцитов мышей.

## Материалы и методы

**Экспериментальные животные.** В исследовании для получения незрелых и зрелых ооцитов использовано 56 самок-доноров мышей линии CD1 в возрасте 2.5 мес. (16 в контроле и 40 в экспериментальной группе) и 6 сте- рильных самцов этой же линии. Животных содержали в клетках с подстилкой из опилок в стандартных условиях конвенционального вивария Института цитологии и ге- нетики СО РАН (Новосибирск, Россия): при комфортной температуре 22–24 °C, свободном доступе к полнорацион- ному сухому гранулированному корму для лабораторных грызунов «Чара» (ЗАО «Ассортимент-Агро», Россия) и очищенной воде, 12:12-часовом цикле дня : ночи. Все эксперименты на животных одобрены Комиссией по био- этике Института цитологии и генетики СО РАН (протокол № 5 от 13.05.2011) и соответствуют Европейской конвен- ции о защите позвоночных животных, используемых для экспериментальных и других научных целей.

**Оценка эффективности диеты.** Cамок-доноров содержали как на стандартной диете (контроль), так и на специализированной, когда дополнительно к обычному корму животным добавляли свиное сало и семена подсолнечника (экспериментальная группа). Пищевые добавки начинали давать с возраста пяти недель. Откормочный эксперимент длился в течение восьми недель. Для подтверждения эф- фективности диеты мышей обеих групп взвешивали перед эвтаназией, а также у самок натощак собирали кровь после декапитации, центрифугировали при 3 250 об/мин (1000 g) в течение 5 мин, после чего собирали плазму и оценивали уровень холестерина и триглицеридов с использованием наборов Холестерин-Ново (АО «Вектор-Бест», Россия) и Триглицериды-Ново (АО «Вектор-Бест»), как рекомендо- вано производителем.

**Незрелые ооциты получали** от 10 самок в контрольной и 30 – в экспериментальной группе. Животных на стадии проэструса подвергали эвтаназии при помощи декапита- ции. Яичники извлекали и измельчали в среде Flushing Solution (FertiPro, Бельгия). Выделенные КОК оценивали под стереомикроскопом Leica S8 APO с увеличением ×80 (Leica Microsystems, Германия). Для исследования брали только те КОК, в которых было не менее пяти слоев ку- мулюсных клеток, плотно прилегающих к прозрачной оболочке ооцита (Hillier et al., 1985). Если КОК имели существенные дефекты, то их отбраковывали.

**Получение стерильных самцов.** Стерилизацию сам- цов проводили путем вазэктомии не менее чем за две недели до начала эксперимента, как было описано ранее (Hogan et al., 1994). Самцов мышей линии CD1 в возрасте шести недель наркотизировали при помощи внутрибрю- шинного введения 0.25 мг/кг препарата медетомедина гидрохлорида (Domitor 1 mg/mL, Orion-Corporation, Финляндия) и через 10 мин – 50 мг/кг препарата золетила (Zoletil, SA, Virbac Sante Animale, Франция). После наркотизации подкожно вводили антибиотик: 0.01 мл амоксициллина тригидрата 150 мг/мл (ОАО «Синтез», Россия). Затем животных помещали на подогреваемый столик, шерсть в зоне операционного поля сбривали, а кожу обрабатывали 70 % этиловым спиртом. При помощи хирургических ножниц делали горизонтальный надрез кожных покровов мошонки длиной ~5 мм. Подтягивали эпидидимисы к краю хирургической раны и разворачи- вали их так, чтобы были видны семенные канатики. Се- мявыносящие каналы отделяли от сопряженных тканей и пережигали раскаленным пинцетом в двух местах, удаляя участок канала между ними. Эпидидимисы возвращали в первоначальное положение. Затем в рану засыпали 2 мг амоксициллина тригидрата (ОАО «Синтез»). Зашивали надрезы наложением двух швов и обрабатывали их Ра- носаном (ООО «Апи-Сан», Россия).

**Получение зрелых ооцитов.** Для выделения зрелых ооцитов проводили стерильное спаривание с использова- нием шести вазэктомированных самцов. В эксперименте участвовало 16 самок (6 в контроле и 10 в эксперимен- тальной группе). Зрелые ооциты выделяли через 20–22 ч после стерильного спаривания. С этой целью выполняли эвтаназию самок-доноров при помощи декапитации, извлекали яичники с прилегающими к ним яйцеводами. Органы переносили в питательную среду M2 (Merck, Германия), разрезали ампулярную часть яйцевода и из- влекали зрелые ооциты. Для удаления кумулюсных клеток использовали гиалуронидазу (Merck) в концентрации 80 ME/мл (Brinster, 1971).

Определение степени ненасыщенности липидов. Методом рамановской спектроскопии (комбинационного рассеяния света – КРС) было выполнено сравнение не- насыщенности липидов в незрелых и зрелых ооцитах. Для исследования изменений в степени ненасыщенности липидов измеряли спектры КРС в диапазоне от 1000 до 3000 см^–1^. Для каждого ооцита измеряли от 30 до 60 спек- тров КРС от разных локальных областей клетки. Латераль- ный и продольный размеры областей, от которых измеряли комбинационное рассеяние света, составляли ~1 и 10 мкм соответственно. Для каждого отдельного набора данных с использованием метода главных компонент выделяли вклад липидов, аналогично подходу, применявшемуся ранее на эмбрионах мыши (Okotrub et al., 2017). Для ха- рактеризации ненасыщенности углеводородных цепочек липидов исследовали соотношение интенсивностей пиков КРС, относящихся к валентным колебаниям двойных С=С связей (~1660 см^–1^) и симметричным валентным колебани- ям метиленовых групп (2850 см^–1^). Ооциты переносили в стеклянный контейнер с лункой, глубиной 300 мкм в капле среды KSOM (Merck), покрывали тонким листом слюды и герметизировали. Измерения осуществляли с исполь- зованием лабораторной экспериментальной установки, состоящей из модифицированного микроскопа (Orthoplan, Leitz, Германия) и решеточного монохроматора SP2500i (Princeton Instruments, Trenton, NJ, США), оснащенного многоканальным детектором Spec-10:256E/LN (Princeton Instruments). Точность определения абсолютной частоты КРС была более чем 1 см^–1^; спектральное разрешение составляло 2.5 см^–1^. Для возбуждения КРС использовали излучение твердотельного лазера (Excelsior, Spectra Physics, США) с длиной волны 532.1 нм.

**Оценка общего количества липидов ооцитов.** Оценка изменения внутриклеточного состава липидов в ооцитах мышей после содержания самок-доноров на двух диетах проведена при помощи окрашивания флуорохромом нильским красным – Nile Red Staining Kit (Merck) с последующей конфокальной лазерной сканирующей микро- скопией (КЛСМ). Метод подробно описан ранее (Romek et al., 2011), при этом нами внесены изменения. Ооциты перед исследованием были зафиксированы в 4 % парафор- мальдегиде (Merck) на фосфатном буфере – PBS (Merck) в течение двух часов, затем трижды отмыты в 50 мкл PBS с содержанием одного мг/мл поливинилпирролидона (Merck) по 5 мин каждая. Стоковый раствор флуорохрома Nile Red (1 мг/мл) был приготовлен посредством разведения красителя в диметилсульфоксиде. Перед окрашиванием стоковый раствор разводили до рабочей концентрации 10 мкг/мл. Ооциты инкубировали в рабочем растворе в течение трех часов при 37 °C, чтобы добиться максимальной интенсивности окрашивания внутрикле- точных липидов, как описано ранее (Genicot et al., 2005). Материал в капле PBS монтировали на предметные стекла.

Изображения образцов получали с помощью инвер- тированного конфокального лазерного сканирующего микроскопа LSM 780 NLO Axio Observer Z1 (Zeiss, Герма- ния) с применением программного обеспечения Zen 2012 (Black Edition) (Zeiss). Все образцы фотографировали объективом Plan-Apochromat ×20 (0.8 NA), возбуждение флюорохрома проводили на длине волны 488 нм арго- новым газовым лазером на мощности 0.1 % × 30 мВт ≈ 30 мкВт, главное дихроичное зеркало было выбрано на 488 нм. Разрешение детектора 512 × 512 пикселей, с задержкой на каждый пиксель в 3.15 мкс. Для детекции сигнала использовали GaAsP детектор (Gallium Arsenic Phosphorus). Полная трехмерная визуализация ооцитов была выполнена с помощью опции Z-stack, установлен- ной на толщину оптического среза в 2.5 мкм. Спектры производили на длинах волн 494–687 нм, с шагом в 9 нм. Число оптических срезов – 40, общая толщина всех опти- ческих срезов – 100 мкм. Все изображения были созданы детектированием в режиме счета фотонов. Итоговое изображение представляет из себя трехмерную матрицу, где для каждого элемента имеется информация о числе детектированных фотонов (абсолютное число фотонов – а. ч. ф.). Для вычитания фона делали три изображения на стекле, где отсутствует материал, при тех же условиях, что были описаны выше, с последующим вычислением среднего. Все оптические срезы (как образца, так и фона) суммировали с применением скрипта для ImageJ, чтобы сформировать итоговое изображение. Для вычитания фоновой флуоресценции использовали Python 3.8 с библиотекой OpenCV.

**Статистический анализ.** Данные анализировали посредством языка программирования R 3.6.2 и с графиче- ской оболочкой RStudio Desktop 1.1.463. Анализ на нор- мальность распределения данных проводили критерием согласия Андерсона– Дарлинга, с учетом коэффициентов эксцесса и асимметрии. Корреляцию между массой тела и уровнем холестерина в крови самок-доноров ооцитов, а также между массой тела самок-доноров ооцитов и аб- солютным числом фотонов оценивали с использованием ранговой корреляции Стьюдента. Значения представлены как среднее ± SEM. Различия средних между группами оценивали t-критерием Стьюдента, а для неравных дис- персий – критерием Кохрана–Кокса. Уровень значимости принимали при p < 0.05.

## Результаты

Данные по влиянию диеты на массу животных, уровень холестерина и триглицеридов в крови представлены в табл. 1. Установлено, что диета с высоким содержанием липидов статистически достоверно ( p < 0.001) приводит к увеличению веса тела животных по сравнению с контролем. Диета с повышенным содержанием липидов до- стоверно ( p < 0.05) вызывала повышение уровня холесте- рина и триглицеридов в крови по сравнению с контроль-ной группой. Имелась достоверная корреляция (r = 0.68; p < 0.01) между массой тела самок-доноров и уровнем холестерина в их крови.

**Table 1.**
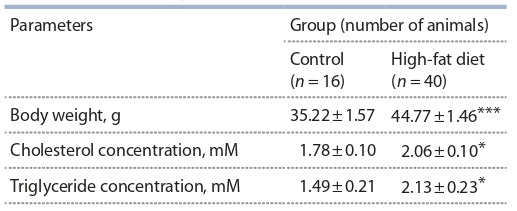
Effects of diet on body weight and concentrations of cholesterol and triglycerides in blood of CD1 mice Differences from the control group are significant at * p < 0.05; *** p < 0.001.

Результаты по влиянию диеты на степень ненасыщен- ности липидов в незрелых и зрелых ооцитах приведены в табл. 2. В ходе исследований не выявлено достоверных различий по степени ненасыщенности липидов в незре- лых и зрелых ооцитах после содержания самок-доноров на двух разных диетах

**Table 2.**
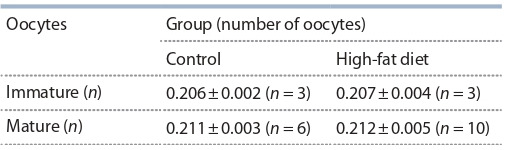
The degree of unsaturation of intracellular lipids in immature and mature oocytes of CD1 mice depending on diet

Влияние диеты на содержание липидов в незрелых и зрелых ооцитах продемонстрировано на рис. 1 и 2. В ре- зультате проведенной работы не выявлено различий по ко- личеству липидов в незрелых ооцитах после содержания самок-доноров на двух разных диетах. Но в зрелых ооци- тах количество липидов было больше ( p < 0.01) в группе мышей, бывших на диете, богатой жирами, по сравне- нию с контролем (8.15 ± 0.37 и 5.83 ± 0.14 млн фотонов соответственно). Кроме того, установлено возрастание количества внутриклеточных липидов у зрелых ооцитов, по сравнению с незрелыми, как у самок, находящихся на стандартной диете (5.83 ± 0.14 и 4.72 ± 0.48 млн фотонов соответственно, p < 0.05), так и у самок, содержавшихся на диете, богатой жирами (8.15 ± 0.37 и 3.45 ± 0.62 млн фотонов соответственно, p < 0.001). Имелась достоверная корреляция (r = 0.91, p < 0.001) между массой тела са- мок-доноров и количеством внутриклеточных липидов в ооцитах.

**Fig. 1.**
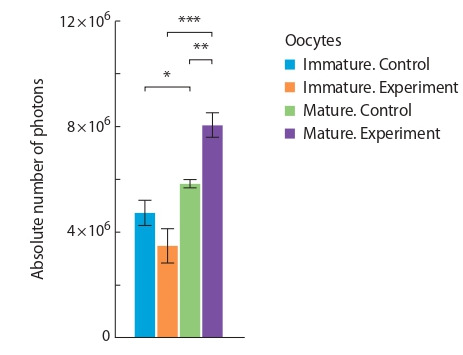
Total fluorescence intensity of lipids in immature and mature oocytes in CD1 mice. * p < 0.05; ** p < 0.01; *** p < 0.001.

**Fig. 2.**
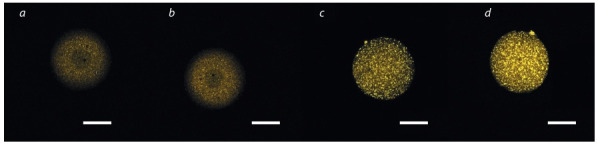
Confocal laser scanning microscopy of immature and mature oocytes of CD1 mice after staining with Nile Red. (a) Immature oocytes, control group; (b) immature oocytes, experimental group; (c) mature oocytes, control group; (d ) mature oocytes, experimental group. Color corresponds to the wavelength of intracellular lipids. Scale bar = 30 μm.

## Обсуждение

В ходе проведенных экспериментов установлено, что диета с повышенным содержанием липидов приводит к увеличению массы тела мышей, а также повышению уровня холестерина и триглицеридов в их крови, что соответствует данным других исследователей (Ma et al., 2012; Li et al., 2018). В настоящее время есть несколько работ, в которых ученые пытаются оценить липидный профиль ооцитов in vivo после влияния диеты (Zeron et al., 2002; Wu et al., 2010; Li et al., 2018). Результаты этих работ достаточно противоречивы.

Используемая нами диета не повлияла на качественный состав липидов в незрелых и зрелых ооцитах мышей от самок-доноров с ожирением. Эти данные могут быть обусловлены тем, что диета была сбалансирована по на- сыщенным и ненасыщенным жирным кислотам, поэтому никаких изменений качественного состава липидов в ооцитах не происходило, как описано ранее в экспери- ментах со сходным дизайном (Dunning et al., 2014; Amstislavsky et al., 2019). Более того, в работе, проведенной на овцах (Zeron et al., 2002), отмечено, что диета, богатая полиненасыщенными жирными кислотами (с добавлени- ем рыбьего жира), влияла на изменение состава внутри- клеточных липидов в клетках кумулюса, но не в незре-лых ооцитах, что может быть связано с высоким содержа- нием в добавке ненасыщенных жирных кислот.

В нашем исследовании обнаружено, что при содержа- нии самок-доноров на диете, богатой жирами, липидов в зрелых ооцитах становилось больше, чем в контроле, т. е. при применении стандартной диеты. Между тем в незрелых ооцитах не было различий по общему количеству липидов при содержании мышей на разных диетах. Некоторые исследования на мышах отмечают воз- растание количества липидов в незрелых ооцитах при содержании мышей на жирной диете (Wu et al., 2010). Однако в недавно опубликованной работе (Li et al., 2018) продемонстрировано возрастание числа крупных липид- ных гранул в клетках кумулюса, но не в самих незрелых ооцитах. Возможно, наблюдаемое в нашем эксперименте отсутствие повышения общего уровня липидов в незрелых ооцитах после специальной обогащенной жирами диеты связано с тем, что эти липиды уходят в другие клетки, в частности кумулюса, в то время как на последующей стадии развития происходит накопление липидов непо- средственно в ооцитах.

Противоречия в выводах описанных выше работ, вы- полненных разными группами исследователей, могут быть связаны с различиями между линиями, а также с тем, что употреблялись корма, отличающиеся по своему составу. В целом можно сказать, что, в отличие от многочисленных работ, в которых используется модель изменения качественного и количественного состава липидов в репродуктивных клетках, культивируемых in vitro (Брусенцев и др., 2019; Amstislavsky et al., 2019), ана- логичные эксперименты в условиях in vivo сопряжены с воздействием большого числа различных факторов, что, по-видимому, и приводит к разнородности резуль- татов.

Обнаружена динамика возрастания количества липидов от незрелых ооцитов к зрелым, наблюдавшаяся при обеих диетах. Но более выраженной эта зависимость была при диете с повышенным содержанием жира. Действительно, жирные кислоты, имеющиеся в крови матери, ответствен- ны за липидный профиль фолликулярной жидкости, из которой они поступают в ооцит через окружающие его клетки кумулюса (Valckx et al., 2014). Ранее с помощью микро- и спектроскопических методов были исследованы распределение, локализация и размер липидных гранул в процессе созревания ооцитов мышей (Dunning et al., 2014; Bradley et al., 2016). В частности, при использовании когерентного антистоксового комбинационного рассеяния выявлено возрастание размера и числа агрегированных ЛГ при созревании ооцитов мышей как in vivo, так и in vitro (Bradley et al., 2016). Сходная закономерность отмечена в работе на крупном рогатом скоте: полуколичественным методом с применением КЛСМ и флуорохрома BODIPY продемонстрировано достоверное возрастание внутрикле- точных липидов при созревании ооцитов in vitro, хотя при созревании ооцитов in vivo наблюдалась лишь тенденция к такому возрастанию (Collado et al., 2017).

Однако в работе на свиньях показано снижение количества внутриклеточных липидов по мере развития ооци- тов in vivo: на стадии герминального везикула содержание липидов было на 21 % выше, чем у созревших до стадии метафазы второго деления мейоза (Romek et al., 2011). Таким образом, имеются и видовые отличия в изменении содержания внутриклеточных липидов в ходе созревания ооцитов (Romek et al., 2011; Dunning et al., 2014; Bradley et al., 2016; Collado et al., 2017). В частности, у свиней снижение количества жиров в ходе созревания ооцитов, вероятней всего, связано с их активным расщеплением, так как у этого вида животных липиды могут выступать в качестве основного энергетического субстрата (Bradley, Swann, 2019).

## Заключение

Результаты нашего исследования показывают, что уве- личение содержания липидов по мере созревания in vivo ооцитов мышей может быть усилено при содержании самок-доноров на диете, богатой жиром. Есть вероят- ность того, что липиды в большом количестве поступают в ооцит мыши из клеток кумулюса при его созревании (Li et al., 2018). Большее количество липидов в зрелых ооцитах, полученных от самок-доноров, содержавшихся на диете, богатой жирами, по сравнению с контролем, по всей видимости, может быть связано с накоплением энергетического субстрата и особенностями метаболизма ожиревших особей, например неспособностью клетки быстро утилизировать поступающие жиры. Имеются примеры того, что фактический состав жирных кислот в ЛГ ооцитов зависит от рациона матери, который определяет жирные кислоты, доступные для ооцита во время развития яич- ников (Bradley, Swann, 2019).

Таким образом, диета, богатая липидами, приводит к увеличению массы тела самок мышей, а также повышает уровень холестерина и триглицеридов в крови этих животных. Содержание самок-доноров на такой диете не влияет на качественный состав липидов незрелых и зрелых ооцитов. При этом наблюдается повышение общего содержания липидов при созревании ооцитов мышей in vivo. Диета, обогащенная жирами, приводит к большему накоплению липидов в зрелых ооцитах.

## Conflict of interest

The authors declare no conflict of interest.
